# Temporal Synchrony in Bodily Interaction Enhances the Aha! Experience: Evidence for an Implicit Metacognitive Predictive Processing Mechanism

**DOI:** 10.3390/jintelligence13070083

**Published:** 2025-07-07

**Authors:** Jiajia Su, Haosheng Ye

**Affiliations:** 1 School of Educational Science, Jiangsu Second Normal University, Nanjing 211200, China; sujiajia0929@163.com; 2 The Center for Embodied Cognition, Guangzhou University, Guangzhou 510006, China

**Keywords:** metacognitive predictive processing model, body interaction, temporal synchrony, Aha! experience, fNIRS

## Abstract

Grounded in the theory of metacognitive prediction error minimization, this study is the first to propose and empirically validate the mechanism of implicit metacognitive predictive processing by which bodily interaction influences the Aha! experience. Three experimental groups were designed to manipulate the level of temporal synchrony in bodily interaction: Immediate Mirror Group, Delayed Mirror Group, and No-Interaction Control Group. A three-stage experimental paradigm—Prediction, Execution, and Feedback—was constructed to decompose the traditional holistic insight task into three sequential components: solution time prediction (prediction phase), riddle solving (execution phase), and self-evaluation of Aha! experience (feedback phase). Behavioral results indicated that bodily interaction significantly influenced the intensity of the Aha! experience, likely mediated by metacognitive predictive processing. Significant or marginally significant differences emerged across key measures among the three groups. Furthermore, fNIRS results revealed that low-frequency amplitude during the “solution time prediction” task was associated with the Somato-Cognitive Action Network (SCAN), suggesting its involvement in the early predictive stage. Functional connectivity analysis also identified Channel 16 within the reward network as potentially critical to the Aha! experience, warranting further investigation. Additionally, the high similarity in functional connectivity patterns between the Mirror Game and the three insight tasks implies that shared neural mechanisms of metacognitive predictive processing are engaged during both bodily interaction and insight. Brain network analyses further indicated that the Reward Network (RN), Dorsal Attention Network (DAN), and Ventral Attention Network (VAN) are key neural substrates supporting this mechanism, while the SCAN network was not consistently involved during the insight formation stage. In sum, this study makes three key contributions: (1) it proposes a novel theoretical mechanism—implicit metacognitive predictive processing; (2) it establishes a quantifiable, three-stage paradigm for insight research; and (3) it outlines a dynamic neural pathway from bodily interaction to insight experience. Most importantly, the findings offer an integrative model that bridges embodied cognition, enactive cognition, and metacognitive predictive processing, providing a unified account of the Aha! experience.

## 1. Introduction

### 1.1. Problem Awareness: Can the Aha! Experience Be Explained by the Metacognitive Predictive Processing Model?

The Aha! moment, a hallmark of creative thinking, is typically described as a sudden cognitive–emotional experience characterized by abruptness, certainty, and a surge of positive affect (Duncker 1945; Gick and Lockhart 1995; Danek et al. 2014). It is not only intellectually captivating but also often regarded as a qualitative leap in problem-solving. Despite widespread recognition of its significance, the underlying mechanism of insight remains theoretically contested. Recent developments suggest a shift in perspective—insight is increasingly viewed as a phenomenon arising from predictive processing bias, rooted in metacognitive prediction errors. This marks the emergence of a new theoretical orientation: the metacognitive predictive processing turn (Becker et al. 2023).

#### 1.1.1. From Representational Change to Metacognitive Predictive Processing: A Theoretical Shift in Understanding Aha! Experiences

Traditionally, the Aha! experience has been explained by the representational change model, which posits that insight occurs when an individual restructures the problem space by breaking free from prior cognitive constraints (Sternberg and Davidson 1995; Ohlsson 2011; Knoblich et al. 1999). While this model effectively accounts for how structural shifts in problem representation can lead to sudden solutions, it primarily emphasizes changes in cognitive content. It provides little insight into the phenomenological dimensions of insight, such as its suddenness, emotional salience, and compelling sense of certainty. As a result, it struggles to answer the following questions: Why does insight feel so sudden? and Why is it accompanied by intense pleasure and confidence? In essence, the representational change model—as a static structural account—falls short of capturing the rich, multidimensional phenomenology of insight experiences, particularly their abruptness, unpredictability, and reward value (Danek and Wiley 2017; Skaar and Reber 2020; Danek 2023).

This has prompted a theoretical shift toward metacognitive perspectives. Metacognition concerns the individual’s awareness, monitoring, and regulation of their own cognitive processes (Flavell 1979). In the context of insight, individuals are not merely solving a problem—they are simultaneously assessing whether the problem is solvable, estimating the time required, and experiencing a form of reflective awareness once the solution is reached. This awareness constitutes a metacognitive judgment, reflecting the ongoing internal modeling and updating of one’s cognitive state (Martinez 2006; Ackerman and Thompson 2017).

As a core component of creativity, insight is also amenable to interpretation within the predictive processing framework. This model suggests that creativity itself can be understood as a form of predictive processing: individuals generate predictions about potential solutions, simulate outcomes internally, and iteratively refine their predictions. Supporting this view, Valtulina and De Rooij (2019) found that adolescents engaged in creative tasks frequently switch between concepts and idea combinations based on predictive evaluation. The frequency of these predictive–evaluative cycles was significantly associated with creative output, indicating that creativity entails dynamic prediction mechanisms.

From the perspective of enactive cognition, the evolutionary function of creativity lies in enhancing the efficiency of interaction and coupling between the individual and their environment. To further optimize this efficiency, humans have developed a metacognitive system that monitors this coupling. When interaction is highly effective, metacognition signals this through reward experiences, such as the well-documented flow state or peak experience. Whether this optimal coupling is achieved is monitored by a core mechanism: the minimization of temporal prediction error at the metacognitive level.

Against this background, the predictive processing model (Friston 2010; Sengupta et al. 2016)—a hierarchically nested cognitive architecture—has provided fertile ground for a subframework known as the metacognitive prediction error model, which has recently been applied to insight research (Dubey et al. 2021). This model proposes that Aha! experiences are triggered when the individual’s prediction about the time or difficulty of solving a problem sharply diverges from the actual outcome—what is referred to as a temporal prediction error. For example, if a problem initially perceived as difficult is solved unexpectedly quickly, this discrepancy generates a strong sense of confidence and positive emotion, thereby producing the Aha! experience.

This framework signals the third major paradigm shift in insight research: from representational change to metacognitive evaluation to metacognitive predictive processing. Crucially, the Aha! experience is not defined by what content was restructured, but by how unexpectedly and rapidly the restructuring occurred. This element of surprise—rooted in processing efficiency—is at the very heart of insight. Capturing and explaining this mechanism is precisely where the predictive processing model demonstrates its theoretical strength.

#### 1.1.2. The Predictive Processing Model as a Hierarchically Nested System: Theoretical Advantages for Explaining Aha! Experiences

In recent years, the Predictive Processing Model (PPM) has emerged as one of the leading frameworks for understanding cognitive function in the brain. According to this model, the brain is not a passive recipient of sensory input but an active “prediction engine” that continuously generates and updates hierarchical models of both the external world and internal states. Through recursive comparisons between top–down predictions and bottom–up sensory input, the brain minimizes prediction errors (PEs) to optimize perception, action, and cognition (Friston 2010; Clark 2013). A key advantage of the PPM lies in its unified computational architecture, which enables cross-level integration—from low-level perception to high-level reasoning, from attentional regulation to emotional experience—making it particularly suitable for explaining the multifaceted phenomenology of insight, or the Aha! experience.

In the context of insight research, the PPM excels at accounting for core features of the Aha! experience, such as suddenness, surprise, pleasure, and certainty. The metacognitive prediction error model (PEmeta) proposed by Dubey et al. (2021) posits that Aha! moments are triggered by a mismatch between an individual’s predictions regarding task difficulty or required time and the actual resolution outcome. When a problem anticipated to be difficult is solved unexpectedly quickly, this discrepancy induces a strong subjective feeling of abrupt emergence and positive effect. The hierarchical structure of the PPM offers a mechanistic explanation: while high-level systems generate predictions such as “I can’t solve this yet” or “this will take longer,” lower-level processes—such as semantic activation or associative retrieval—may suddenly arrive at the solution. This results in a bottom–up violation of the higher-order prediction. When such top–down predictions are rapidly overturned, a surge of prediction error signals ascends the hierarchy, triggering belief revision and reward feedback, which in turn give rise to the co-occurrence of surprise, joy, and cognitive certainty (Sengupta et al. 2016; Friston et al. 2017).

Empirical evidence has increasingly supported this framework. Moroshkina et al. (2024) demonstrated that the intensity of the Aha! experience is significantly correlated with the decrease in subjective difficulty ratings before and after problem solving, lending support to the “difficulty prediction error” hypothesis. Becker et al. (2024) further showed that the experience of surprise is particularly intense when participants incorrectly predict a problem to be unsolvable but end up solving it. Similarly, Stuyck et al. (2021) found that participants tend to retrospectively underestimate the original difficulty of problems associated with Aha! experiences, reinforcing the idea of underlying prediction bias. Complementary to this, the processing fluency theory suggests that sudden ease of cognitive processing makes individuals more prone to experiencing insight (Topolinski and Reber 2010).

At the neural level, Aha! Experience are often accompanied by activation in reward-related brain regions, particularly the ventral striatum and dopaminergic circuits (Kizilirmak et al. 2016; Tik et al. 2018; Oh et al. 2020). These activations resemble the reward signals observed in reinforcement learning models driven by positive prediction errors (Sutton and Barto 2018; Den Ouden et al. 2012). Becker et al. (2023) further highlighted that the greater the prediction error, the stronger the reward system activation, and the more intense the associated feelings of pleasure and confidence—suggesting that multiple dimensions of insight may be jointly mediated by a unified prediction error mechanism.

#### 1.1.3. The Challenge of Insight: How Does the Body Influence the Aha! Experience Through Implicit Metacognitive Predictive Processing?

While the prediction error framework has successfully incorporated the Aha! experience into the domain of metacognitive processing—arguing that it originates from a mismatch between expected and actual cognitive performance (Dubey et al. 2021; Moroshkina et al. 2024)—empirical investigations of this model remain predominantly grounded in the explicit level of metacognition. That is, they rely heavily on individuals’ subjective reports and conscious evaluations, such as estimates of solution time or confidence in correctness. However, two hallmark features of insight—its suddenness and unpredictability—are difficult to explain solely through consciously accessible, deliberative assessments.

Multiple studies suggest that metacognition comprises two distinct levels: explicit and implicit (Dienes and Perner 2002; Petty and Briñol 2006; Gawronski and Bodenhausen 2006). Frith (2012) was among the first to propose, in the context of social interaction, that explicit metacognition involves conscious monitoring and verbal expression, whereas implicit metacognition likely regulates cognitive states in real time beneath the threshold of awareness through bodily signals, processing fluency, and automatic adjustment mechanisms. Further research indicates that implicit metacognition not only exists, but also relies heavily on the perception and evaluation of bodily signals such as heartbeat, respiration, and interoception. It represents a form of metacognitive monitoring that is difficult to verbalize, yet plays a vital role in regulating cognition and emotion (Herbert and Pollatos 2012; Häfner 2013; Skulmowski and Rey 2017; Faivre et al. 2020). Interestingly, in the domain of insight research, Laukkonen et al. (2021) found that Aha! Experience are often accompanied by spontaneous bodily reactions, further supporting the idea that implicit metacognition can be expressed and modulated through bodily signals.

Research in embodied cognition aligns strongly with this view of implicit metacognition. It suggests that cognition is not confined to brain-bound computation, but rather emerges from the dynamic interaction among the brain, body, and environment (Gallagher 2005; Ye 2011; Varela et al. 1991). Empirical studies have further demonstrated that predictive processing is inherently embodied. For example, Bitu et al. (2021) showed that participants drawing on a touchscreen exhibited greater originality than those drawing on paper—underscoring the role of bodily feedback in creative cognition and offering indirect support for predictive processing models grounded in sensorimotor engagement.

From this perspective, we propose that the body’s influence on Aha! experiences operates via an implicit metacognitive mechanism of temporal prediction error minimization. This notion is further supported by empirical evidence. Laukkonen et al. (2021) found that subtle changes in handgrip strength during insight tasks could predict the intensity of the Aha! experience, suggesting that insight is not purely a cognitive event but reflects a body–mind coupling process. Additionally, Gordon et al. (2023), through large-scale fMRI analyses, introduced the Somato-Cognitive Action Network (SCAN), which revealed strong overlap between motor cortex regions and higher-order cognitive functions—further supporting the embodied nature of insight.

Taken together, these findings suggest that the generation of insight can be conceptualized as a process of implicit temporal prediction error minimization grounded in bodily systems. Under this framework, the Aha! experience is not merely the cognitive restructuring of content, but a sudden event triggered by prediction error, embodied in physiological states, and reinforced by belief revision and affective feedback. This theoretical perspective offers a robust foundation for future empirical work—particularly studies examining how bodily interactions modulate insight.

### 1.2. How Bodily Interaction Influences the Aha! Experience: The Experimental Potential of the Mirror Game Paradigm

From the perspective of implicit metacognition, the suddenness of the Aha! experience is not merely rooted in prediction errors within the cognitive system concerning problem-solving success. Rather, it may be driven by a coupling tension among the bodily, cognitive, and emotional systems during interaction. Against this backdrop, identifying an experimental paradigm that both activates embodied interaction and captures dynamic predictive processing states in real time offers a promising breakthrough in insight research. The Mirror Game, a paradigm characterized by its quantifiability, interactivity, and bodily coordination demands, presents a compelling methodological bridge between bodily engagement and cognitive transformation.

#### 1.2.1. The Mirror Game as a Canonical Experimental Paradigm

The Mirror Game is an experimental paradigm centered on real-time bodily interaction, first introduced by Noy et al. (2011) to study how interpersonal movement synchrony arises naturally in the absence of verbal communication. In its basic form, two participants face each other and engage in upper-body movement imitation tasks, attempting to achieve maximum synchrony without using verbal or nonverbal cues. The paradigm consists primarily of two modes: (1) the Leader–Follower condition, where one participant initiates movement and the other imitates; and (2) the Joint Improvisation condition, where both participants co-create movements with no fixed leader. Using precise motion-tracking technologies, this paradigm captures key indicators of interaction quality—such as spatial synchrony, temporal precision, velocity variability, and movement complexity—enabling fine-grained quantification of bodily coordination.

One of the paradigm’s core strengths lies in its ability to monitor interactive movement with high spatial and temporal resolution. Algorithmic analysis allows for the accurate quantification of bodily interaction through metrics such as movement synchrony, temporal coordination, velocity variability, and complexity of motion. This capacity for objective measurement addresses methodological limitations common in earlier embodied metaphor research, such as subjectivity and low replicability, thereby providing a robust data foundation for investigating mechanisms of bodily interaction.

More importantly, the Mirror Game offers an operable model of “bodily–cognitive synchrony.” Empirical studies have shown that this paradigm elicits enhanced interpersonal closeness (Noy et al. 2015) and creative expressiveness (Gueugnon et al. 2016), underscoring its ecological validity for studying higher-order cognitive phenomena, particularly those involving creativity and affective connection.

Additionally, unlike traditional paradigms that rely on static postures or individualized tasks, the Mirror Game emphasizes real-time dynamics, bidirectional interaction, and situational embeddedness. As such, it represents a paradigmatic shift within embodied cognition research—from static representation to interactive process construction (Gallagher 2005; Di Paolo and De Jaegher 2012).

#### 1.2.2. The Experimental Potential of the Mirror Game Paradigm for Investigating How Bodily Interaction Influences the Aha! Experience

Within the framework of the metacognitive predictive processing model, the Aha! experience is conceptualized as the subjective release of a higher-order prediction error, that is, the individual holds certain expectations about the difficulty, duration, or likelihood of solving a problem, which are then unexpectedly violated by a rapid solution, triggering a sudden emotional shift (Dubey et al. 2021; Moroshkina et al. 2024). However, to further account for the characteristic “suddenness” and “ineffability” of insight phenomena, it is necessary to extend the predictive processing framework to the level of implicit metacognition—nonverbal, automatic predictive pathways based on bodily sensations, temporal perception, and emotional states (Laukkonen et al. 2021). We propose that the Mirror Game, as a highly interactive and synchronous embodied paradigm, holds substantial promise as an experimental platform for insight research. Its influence can be theoretically understood through two key dimensions: implicit metacognitive processing and metacognitive temporal prediction error.

First, regarding implicit metacognitive processing, the Mirror Game activates sensorimotor exchanges involving bodily movement, partner feedback, and rhythmical coordination, thereby eliciting a range of embodied metaphorical experiences. For instance, during sustained movement synchronization, individuals may experience states such as “alignment,” “coordination,” “resistance,” or “disconnection.” Although nonverbal, these sensations can activate metaphorical schemas relevant to problem solving—such as “the idea clicked,” “I’m stuck,” or “a breakthrough occurred.” Keefer et al. (2014) proposed the “metaphoric fit hypothesis,” which posits that bodily states can automatically activate corresponding metaphorical conceptual structures, thereby influencing cognitive strategies and performance in abstract tasks. These embodied metaphors are recognized as important channels for restructuring cognitive representations, influencing problem-solving strategies and attentional resource allocation through automatic, nonconscious mechanisms. Due to the continuous and dynamic nature of bodily feedback and interactive rhythm in the Mirror Game, the generation of such metaphors is temporally sensitive, aligning closely with the abrupt and affect-laden characteristics of insight moments.

Second, in terms of metacognitive temporal prediction error, the Mirror Game possesses a unique capacity to induce subjective temporal expansion or illusions of temporal co-presence (Hart et al. 2014). In highly synchronous interactions, participants tend to underestimate the passage of time or overestimate their sense of control and efficiency. Such temporal distortions may lead to the metacognitive expectation that the problem-solving task is ongoing. When the solution then suddenly emerges, the large discrepancy between expected and actual task duration forms a potent prediction error, triggering the characteristic surprise and pleasure associated with the Aha! experience (Dubey et al. 2021). Additionally, the social nature of the Mirror Game activates mechanisms of empathy and intention inference, whereby participants form implicit expectations about task difficulty, emotional tone, or likelihood of success through nonverbal cues such as facial expressions or motion feedback. These nested emotional and social signals provide additional dimensions of input into the predictive system, enhancing the salience of the resulting insight.

In summary, the Mirror Game paradigm provides a novel methodological pathway for modeling the Aha! experience by activating implicit metacognitive mechanisms (e.g., embodied metaphor generation) and modulating temporal perception and social expectations (thereby amplifying temporal prediction error). It constitutes a pre-reflective and pre-verbal channel for cognitive regulation, while also serving as an interactional interface compatible with predictive processing theory. In the present study, we integrate the Mirror Game into an insight research paradigm to investigate how bodily interaction along the temporal dimension (Immediate vs. Delayed vs. No Mirror Game) modulates the generation of prediction error during insight. This approach aims to provide foundational empirical support for applying embodied cognition theory to the domain of insight experiences.

### 1.3. A Novel Experimental Paradigm of Metacognitive Predictive Processing for Investigating the Mechanism Linking Bodily Interaction and Insight

In terms of experimental manipulation of metacognitive time prediction error, Dubey et al. (2021) were the first to empirically validate its association with insight through a series of large-scale, preregistered studies. They divided the insight task into three phases—time prediction, problem solving, and insight evaluation—and found that minimizing temporal prediction error plays a critical role in the generation of insight. Building on this paradigm, Moroshkina et al. (2024) introduced a subjective difficulty rating, showing that the intensity of insight was significantly correlated with the discrepancy between predicted and actual difficulty. This suggests that the perception of a problem being “easier than expected” can enhance the Aha! experience. Becker et al. (2024) further manipulated perceived solvability and found that when participants incorrectly predicted a problem to be unsolvable but then succeeded, they reported significantly stronger insight. This highlights the key role of negative prediction error and the cognitive reversal it induces. Collectively, all three studies adopted the core strategy of explicitly separating prediction from actual processing, and by manipulating expectations of time, difficulty, and solvability, they effectively captured the dynamic role of metacognitive predictive processing in insight experiences.

Following this rationale, the present study employed a between-subjects design with a single experimental factor: temporal synchronization in bodily interaction, which was divided into three levels. (1) Immediate Group. During the learning phase, participants learned six movements from the experimenter (two in each of the vertical, horizontal, and sagittal planes), and in the Mirror game phase, they immediately mimicked the experimenter’s actions in real time. (2) Delayed Group. Participants learned the same six movements, but during the Mirror game phase, they were instructed to wait for three seconds after observing each of the experimenter’s movements before initiating imitation. This delay was based on the “three-second time window” theory (Pöppel 2004), which posits that human cognitive processing operates in temporal units of approximately three seconds, and has been applied in synchrony experiments to define delay durations (Matsuda et al. 2015). (3) Control Group (No Mirror Game). Participants did not engage in either the learning or Mirror game tasks.

The core insight task is broken into three phases aligned with the predictive processing model: prediction (anticipated solution time), action (actual solution time), and evaluation (intensity of the insight experience). The dependent variables include the following: (1) Predicted Solution Time: Participants estimate the time needed to solve each of 10 riddles (in seconds or minutes). (2) Time Prediction Error: After each solution, participants indicate whether the actual time taken was shorter, longer, or equal to their prediction. (3) Insight Intensity: For each riddle solved with an insight experience, participants rate the strength of the Aha! experience on a 1–7 Likert scale. Additional behavioral measures include the following: (1) Number of riddles solved within a 10-min limit. (2) Accuracy rate of solutions. (3) Total number of Aha! experiences reported. (4) Five-dimensional evaluation of insight (pleasantness, suddenness, surprise, certainty, impasse), rated on a 1–5 Likert scale.

In terms of neural mechanisms, Gordon et al. (2023) proposed the Somato-Cognitive Action Network (SCAN), identifying overlapping regions in the motor cortex that are involved not only in movement execution but also in abstract cognition. Their findings—based on the largest fMRI datasets to date—suggest that motor systems may underpin higher-order cognition, including creativity and insight. This network may offer a additional major line of neural evidence for embodied cognition. Consequently, if insight occurs within the motor system, it would lend neurophysiological support to the embodied nature of insight and creativity. In the present study, neurophysiological indicators focus on the SCAN-related brain regions, with dependent variables including the following: (1) Amplitude of Low-Frequency Fluctuations (ALFF). (2) Channel-Level Functional Connectivity (CH-FC). (3) Network-Level Functional Connectivity (NW-FC).

We hypothesize that higher synchrony in bodily interaction will provide stronger rhythmic feedback, enhancing individuals’ ability to accurately predict task efficiency in temporal terms. This should result in larger positive prediction errors (i.e., tasks are solved faster than predicted), thereby triggering insight experiences. Conversely, delayed interaction may disrupt rhythm and reduce the likelihood of insight. As the SCAN network represents a potential neural substrate of embodied cognition, we expect greater neural activation and connectivity in the synchronous condition, supporting the integrated mechanism of bodily interaction to prediction error to insight.

**H1 (Behavioral** **Hypothesis):** *Bodily synchrony in the mirror game is expected to significantly enhance the sensitivity of metacognitive predictive processing, thereby increasing both the likelihood and intensity of insight experiences.*

Specifically, compared to the Delayed and Control Groups, participants in the Immediate Mirror Game Group are anticipated to exhibit shorter predicted solution times, larger positive prediction errors (i.e., actual solution times significantly shorter than expected), and a higher number of reported insight experiences. These effects suggest that synchronous interaction facilitates the emergence of insight by amplifying efficiency expectations and prediction discrepancies.

**H2 (Neural** **Hypothesis):** *Temporal synchrony in bodily interaction during the mirror game is hypothesized to modulate the neural basis of insight by enhancing activity and connectivity within the SCAN.*

Compared to the Delayed and Control Groups, the Immediate Mirror Game Group is expected to show higher Amplitude of Low-Frequency Fluctuations (ALFF) and stronger Functional Connectivity (FC) within SCAN-related brain regions during the insight task. These neural indicators collectively reflect the regulatory influence of bodily synchrony on the predictive processing system underlying insight generation.

## 2. Experimental Procedure

### 2.1. Participants

Based on the “metacognitive prediction error minimization” framework proposed by Dubey et al. (2021), who first established an exploratory paradigm for studying the “Aha! experience” using metacognitive predictive processing and computational modeling, this study further adapts the paradigm to examine how time synchronization in bodily interaction influences the metacognitive predictive processing of the “Aha! experience.” Consequently, no prior studies have conducted experiments using this adapted paradigm. To determine the appropriate sample size, we conducted an a priori power analysis using G*Power 3.1.9.7. Assuming a large effect size (f = 0.5), a significance level of α = 0.05, and a desired statistical power of 0.80, the analysis indicated that a one-way ANOVA with three independent groups would require a total sample size of 42 participants, approximately 14 per group. Given the time-intensive nature of embodied cognition studies, especially those involving functional near-infrared spectroscopy (fNIRS), this sample size is considered reasonable for an initial exploratory study.

During the pilot phase, 13 students from Guangzhou University participated in the study, randomly assigned to one of three groups, with 3 participants in each group. As the behavioral and fNIRS experimental protocols were not yet fully stabilized during this phase, the data from these participants were excluded from further analysis.

In the formal experimental phase, 69 undergraduate students from Guangzhou University participated in the study: 24 participants in the Immediate group, 24 in the Delayed group, and 21 in the No Mirror Game group. Due to issues such as interruptions in the fNIRS equipment connection and participant-related problems, data from 10 participants were incomplete and therefore excluded from further fNIRS data analysis. Ultimately, valid fNIRS data from 59 participants were included: 21 in the Immediate group, 19 in the Delayed group, and 19 in the No Mirror Game group. All participants were in good physical and mental health, had normal or corrected-to-normal vision, were unaware of the experimental content prior to participation, and had no previous experience with similar experiments.

This study was approved by the Ethics Committee of Guangzhou University. All participants read and signed an informed consent form before the experiment. Upon completion of the experiment, each participant received a compensation of CNY 20.

### 2.2. Experimental Design and Procedure

First, the experimenter guided the participants into the laboratory and seated them in pre-assigned chairs. The functional near-infrared spectroscopy (fNIRS) equipment was then fitted onto the participants, and the signals from the source and detector probes were calibrated to ensure sufficient activation channels.

**Figure 1:**
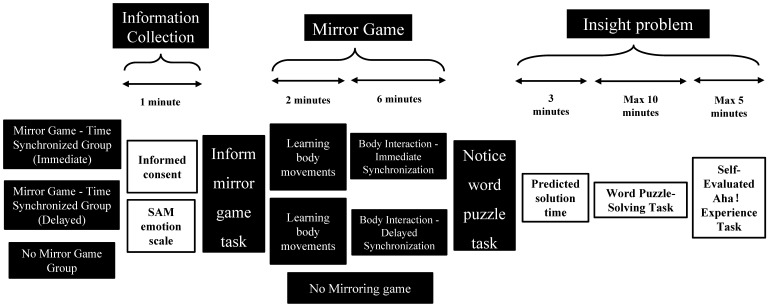
Flowchart of the Experimental Procedure.

The experimental process was divided into three main stages (Figure 1) as follows:

Once the fNIRS equipment was activated, participants were instructed to calm their emotions for one minute. This step served two purposes: helping participants focus on the upcoming tasks and establishing a one-minute long baseline for fNIRS data collection.

#### 2.2.1. Information Collection Stage (Approximately 1 Min)

Each participant was required to read and complete the following documents:

**(1)**  **Informed Consent Form:**

This followed the Declaration of Helsinki to ensure participants’ awareness of their rights regarding the experiment.

**(2)**  **Self-Assessment Manikin (SAM) Scale:**

Participants evaluated their emotional states. If they had any questions, the experimenter provided explanations. After signing and completing the documents, the forms were handed back to the experimenter.

#### 2.2.2. Mirror Game Stage

Participants were informed of the Mirror Game task: The experimenter explained the rules of the Mirror Game. After the experimenter performed a body movement, participants were required to imitate the same movement. The experimenter reminded participants to avoid exaggerated head movements and answered any questions they had.

**(3)**  **Learning Phase (approximately 2 min):**

Participants in the Mirror Game Groups (both immediate and delayed) were taught six body movements by the experimenter, covering up–down, left–right, and forward–backward dimensions (two movements for each dimension). Participants were allowed to practice, and the experimenter corrected them as needed.

**(4)**  **Formal Experimental Phase (6 min):**

The experimenter randomly selected movements from the six taught in the learning phase. Participants were required to mimic the experimenter’s movements at a specified frequency. In the Immediate Mirror Group, participants immediately performed the same movement; in the Delayed Mirror Group, participants imitated the same movement after a three-second delay.

#### 2.2.3. Insight Problem Stage

Participants were introduced to the Word Puzzle-Solving Task: The Word Puzzle-Solving Task was based on materials provided by Professor Qiu Jiang from Southwest University (Qiu et al. 2006), and the Chinese character chunk deconstruction task provided by Professor Luo Jin from Capital Normal University (Luo 2004; Luo et al. 2006). After testing the difficulty level through pilot experiments and interviewing participants, the character chunk deconstruction task was selected as the experimental material.

Chinese Character Chunk Deconstruction Task: The riddles consist of two Chinese characters. The game rule is to move one or two strokes from the second character to the first character, forming two new, correct characters. The answer is the two newly formed characters.

**(1)**  **Predicting the “Solution Time” (3 min):**

Participants were asked to write down their predicted solution time for the 10 riddles on sticky notes. Each prediction was written on a separate note and placed on the desk. Participants were reminded not to attempt to solve the riddles while predicting, but to quickly estimate the solution time in seconds (s) or minutes (min) (e.g., 1 s, 10 s, 1 min, 2 min). This task lasted 3 min.

**(2)**  **Solving the Riddles (10 min):**

Participants were invited to solve the 10 riddles and write their answers on the corresponding sticky notes, also marking the actual solution time with “>”, “=”, or “<” on each note. The task lasted approximately 10 min.

**(3)**  **Self-Assessment of “Aha! Experience” (about 5 min):**

Participants were asked to evaluate whether they had an Aha! experience for each of the 10 riddles (marking √ if yes) and to rate the Aha! experience on a scale of 1–7, including dimensions such as pleasure, suddenness, surprise, certainty, and impasse (1–5 points). This task took about 5 min.

Note: Both the researcher and participants were in the same room. If participants had any questions, they were encouraged to seek help. Once the Aha! self-assessment was completed, the experimenter stopped the fNIRS data collection and removed the equipment from the participants.

Finally, the experimenter provided participants with the experiment payment registration form. After receiving their CNY 20 payment, the participants were escorted out of the laboratory, and any further questions they had were addressed by the experimenter.

**Figure 2:**
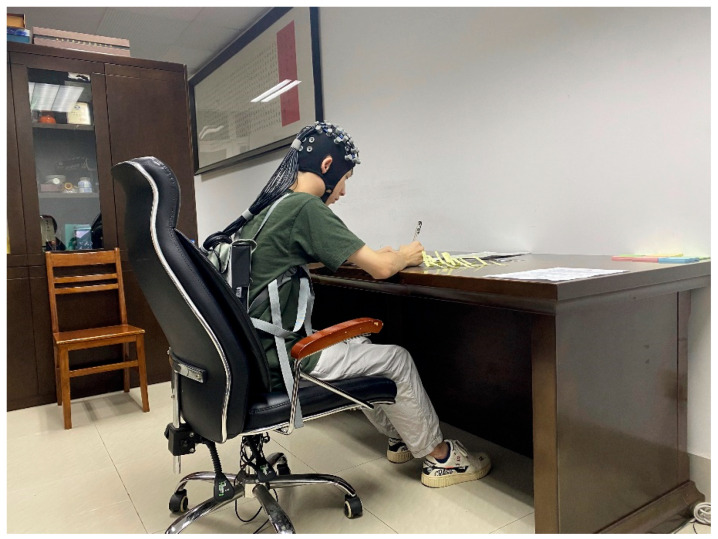
Experimental Setup.

As shown in Figure 2, the participant remained seated with fNIRS equipment attached throughout the task.

### 2.3. fNIRS Data Collection

#### 2.3.1. Experimental Equipment

**Figure 3:**
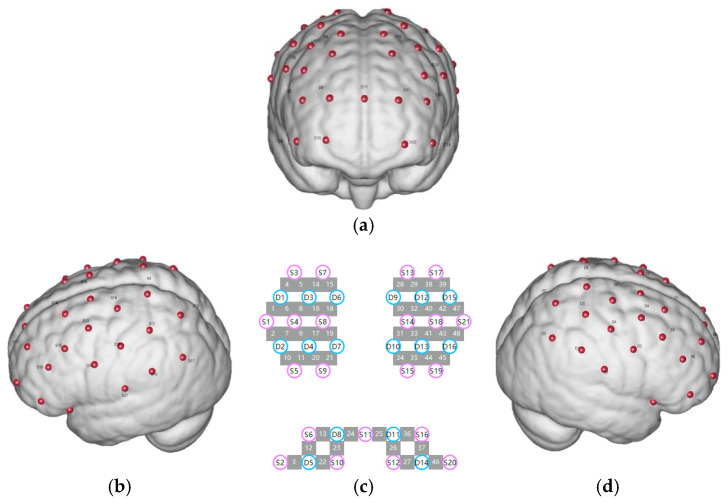
Experimental 3D Arrangement of fNIRS Optodes and Cortical Mapping. (**a**) Top view of the optode distribution over the cortex. (**b**) Left lateral view of the optode arrangement. (**c**) Channel montage and source-detector layout based on the 10–20 system. (**d**) Right lateral view of the optode arrangement. Red dots indicate the fNIRS channels located over prefrontal and motor cortical areas.

In this study, we used the NirSmartII-3000 portable near-infrared brain functional imaging system (manufactured by HuiChuang, Danyang, Jiangsu, China), which operates at two near-infrared wavelengths: 730 nm and 850 nm. The system is equipped with 24 emitters and 16 detectors, allowing for up to 63 channels of data acquisition. The system was employed to measure relative changes in oxygenated hemoglobin (HbO) and deoxygenated hemoglobin (HbR) concentrations, with neural activity being inferred based on light intensity signals converted through the modified Beer–Lambert law. The sampling rate was set at 11 Hz. For each participant, the optodes consisted of 21 emitters and 16 detectors, forming a total of 48 channels, with an inter-optode distance of approximately 3 cm. The optodes were positioned using the 10/20 EEG system, and virtual localization was employed to align the recording channels with corresponding cortical regions. The primary regions of interest in this study were the prefrontal cortex and the motor cortex (Figure 3).

#### 2.3.2. Identification of Regions of Interest (ROI)

**(1)**  **Are Aha! Experiences and Metacognitive Temporal Prediction Errors Related to the SCAN Network?**

Dubey et al. (2021) proposed a “metacognitive prediction error minimization” framework to explain the occurrence of Aha! experience. This theory suggests that during problem-solving, individuals not only solve the problem but also maintain a metacognitive model of their ability to solve it and estimate the time required. When there is a positive prediction error in metacognition—i.e., when individuals solve the problem much faster than expected—an Aha! experience arises. Through three large-scale pre-registered experiments, this study demonstrated a relationship between metacognitive prediction errors and Aha! experiences.

Bader and Wiener (2021) highlighted that error monitoring is a crucial human ability supporting metacognition. When individuals receive feedback from previous trials, they improve in estimating motor time, indicating metacognitive awareness of their temporal errors. To explore the neural basis of this ability, both male and female participants underwent functional MRI (fMRI) while performing a visual time reproduction task. Neuroimaging data showed dissociations between estimating and reproducing time intervals. The former likely engaged brain regions associated with the default mode network (DMN), including the superior frontal gyrus, occipital cortex, and posterior cingulate cortex. The latter activated regions traditionally linked to the timing network (TN), such as the supplementary motor area (SMA), precentral gyrus, and right supramarginal gyrus. Overall, these findings suggest that the DMN and TN work together to regulate temporal perception and enhance temporal performance. The TN is a neural network involved in temporal tasks such as time estimation, memory, and prediction. Its primary components include the SMA, precentral gyrus, and right supramarginal gyrus.

In another study, Ogawa et al. (2018) conducted an exploratory analysis of resting-state functional connectivity (RSFC) constrained by voxel-based morphometry (VBM). They found that individual scores on an insight test were positively correlated with gray matter volume (GMV) in the right insula, middle cingulate cortex, and frontal pole, while negatively correlated with GMV in the left cerebellar lobule and right SMA. Simply put, insight-related connections involve motor-associated areas, with a negative correlation between the right SMA’s GMV and Aha! experiences. This suggests that the structure of the right SMA may be inversely associated with the ability to generate creative insights, where smaller GMV in this area is linked to fewer insights.

Additionally, as mentioned earlier, Gordon et al. (2023) proposed the “Somato-Cognitive Action Network” (SCAN) in a Nature paper, offering strong neurocognitive evidence supporting embodied cognition beyond the mirror neuron system.

Thus, we speculate that Aha! experiences and metacognitive prediction errors are related to the SCAN network.

**(2)**  **Are Aha! Experiences and Metacognitive Processes Related to the DAN, RN, and VAN Networks?**

The Dorsal Attention Network (DAN), also anatomically known as the dorsal frontoparietal network (D-FPN), is a large-scale brain network. Its core regions include the intraparietal sulcus (IPS) and frontal eye fields (FEF), with additional areas such as the middle temporal area (MT+), superior parietal lobule (SPL), supplementary eye fields (SEF), and ventral premotor cortex. The DAN plays a prominent role in the voluntary direction of visual spatial attention. Initially defined and named by Corbetta et al. in the early 2000s, the network is involved in top–down stimulus and response selection across various modalities (e.g., auditory, tactile) (Corbetta and Shulman 2002). The DAN interacts with the Ventral Attention Network (VAN) during task switching or attentional shifts (Tamber-Rosenau et al. 2018).

The VAN, also known as the ventral frontoparietal network (VFN) or ventral attention system (VAS), is predominantly right-hemisphere-lateralized and involves the temporoparietal junction (TPJ) and ventral frontal cortex. It responds to unexpected salient stimuli (Fox et al. 2006; Farrant and Uddin 2015). The TPJ integrates signals from the thalamus, limbic system, and sensory systems, playing a crucial role in self-other distinction and theory of mind (Abu-Akel and Shamay-Tsoory 2011).

The right TPJ (rTPJ) is involved in processing new stimuli and shifting attention. Neuroimaging and lesion studies by Blakemore et al. (2002) showed that rTPJ processes internal activity signals and external environmental information, impacting social interaction and awareness of bodily signals. Damage to the rTPJ often results in hemispatial neglect, where individuals fail to notice stimuli on the left side. Therefore, we can speculate that the VAN, particularly the rTPJ, is closely tied to metacognitive prediction processing.

Additionally, the brain has evolved a reward system that drives the pursuit of pleasure. Dopamine, a key neurotransmitter, underpins this reward system, which consists of neurons in the ventral tegmental area (VTA) projecting to the nucleus accumbens (NAc), prefrontal cortex (PFC), and amygdala. This system plays a central role in emotion, motivation, and reward. Haber and Knutson (2010) proposed a reward circuit in which the ventral striatum receives input from prefrontal areas, including the orbitofrontal cortex and anterior cingulate cortex, sending output to the ventral pallidum and midbrain, eventually looping back to the prefrontal cortex via the medial dorsal nucleus of the thalamus. Kringelbach (2005) identified the orbitofrontal cortex as the strongest candidate for linking rewards like food to pleasurable experiences. The reward system can thus be seen as a marker for prediction error minimization in the brain (Skvortsova et al. 2014; Corlett et al. 2022).

**Regions of Interest for Experiment: DAN, VAN, and RN Networks.** For the experiment, we primarily investigated the following four brain networks:

**(1)**  **The Somato-Cognitive Action Network (SCAN) (M1 and SMA):** Channels 19, 20, 21, 22, 29, 30, 31, 32, 34, 35, 38, 46, 48.
**(2)**  **The Dorsal Attention Network (DAN) (FEF):** Channels 27, 28, 39, 40.
**(3)**  **The Ventral Attention Network (VAN) (DLPFC):** Channels 3, 8, 13, 14, 17, 18, 23, 24, 25, 26.
**(4)**  **The Reward Network (RN) (FPC):** Channels 4, 5, 6, 7, 15, 16.

## 3. Experimental Results

In this study, traditional statistical analyses of behavioral data were conducted using IBM SPSS Statistics 26. Bayesian analyses and all data visualizations were performed using R, while the fNIRS neuroimaging data were preprocessed and analyzed with Matlab (2017b).

### 3.1. Behavioral Data Statistical Analysis

#### 3.1.1. Primary Variables: Predictive Processing Performance in Aha! Experiences

**(1)**  **Predicted Solution Time: 2 × 3 Weighted Chi-Square Test**

To explore differences in the “composition ratio” of predicted solution times, the dependent variable “predicted solution time” was categorized into two groups: “measured in seconds” and “measured in minutes.” Since the dependent variable became dichotomous, a 2 × 3 weighted chi-square test was performed based on the frequency of the predicted solution time categories.

Descriptive Statistical Analysis Results: The descriptive statistical analysis of the chi-square test for predicted solution time across the three groups shows the frequency and corresponding percentages for the categories “measured in seconds” and “measured in minutes”, as shown in Table 1.

**Figure 4:**
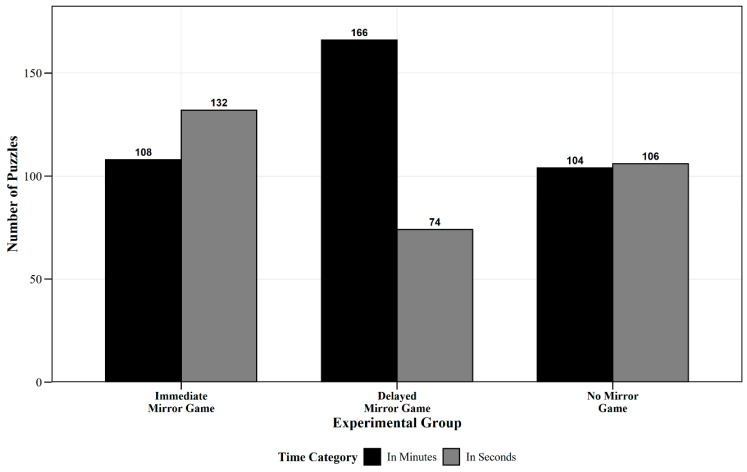
Weighted chi-square test statistical results for predicted solution time. Note: Distribution of predicted solution time categories across experimental groups. Chi-square test results: *χ*^2^ = 32.15, *p* < 0.001. Error bars represent standard error. *p* < 0.05.

Between-Group Comparison Results: Further analysis was conducted using a weighted chi-square test, with group membership (Immediate, Delayed, and No Mirror Game Groups) as the between-group variable, and the classification of predicted solution time (measured in seconds or minutes) as the outcome variable. The results indicated significant differences between the groups: the Immediate Mirror Game group had 55% of predictions in seconds and 45% in minutes, the Delayed Mirror Game group had 30.8% in seconds and 69.2% in minutes, and the No Mirror Game group had 50.5% in seconds and 49.5% in minutes. These differences were statistically significant, *χ*^2^ = 32.15, *p* < 0.001 (Figure 4).

**(2)**  **Time Prediction Error: 2 × 3 Weighted Chi-Square Test**

Similarly, to examine the differences in the composition ratio of time prediction errors, the dependent variable “time prediction error” was categorized by the researchers into three classifications: “Prediction Greater than Actual,” “Prediction Equal to Actual,” and “Prediction Less than Actual.” Since this is a three-category variable, a 3 × 3 weighted chi-square test was conducted, with frequencies weighted according to the selected classification of time prediction errors.

Descriptive Statistical Analysis Results: A descriptive statistical analysis using chi-square tests was performed for the time prediction errors in the three groups, showing the frequencies and corresponding percentages for the categories “Prediction Greater than Actual,” “Prediction Equal to Actual,” and “Prediction Less than Actual”, as shown in Table 2.

**Figure 5:**
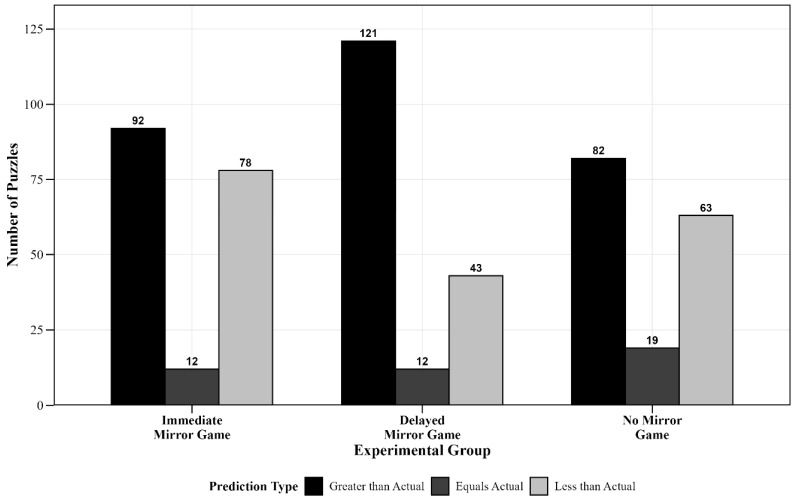
Weighted chi-square test statistical results for time prediction error. Note: Distribution of time prediction error patterns across experimental groups. Chi-square test results: *χ*^2^ = 19.56, *p* < 0.001. Categories represent participant predictions compared to actual solution time.

Group Comparison Results: Further analysis was conducted by treating the experimental groups (Immediate Mirror Game Group, Delayed Mirror Game Group, and No Mirror Game Group) as between-group variables. A weighted chi-square test was applied to the classification of time prediction errors (“Prediction Greater than Actual,” “Prediction Equal to Actual,” and “Prediction Less than Actual”) for the insight riddle-solving task. The results showed significant differences among the groups: the Immediate Mirror Game Group (“Prediction Greater than Actual” 50.5%, “Prediction Equal to Actual” 6.6%, and “Prediction Less than Actual” 42.9%), the Delayed Mirror Game Group (“Prediction Greater than Actual” 68.8%, “Prediction Equal to Actual” 6.8%, and “Prediction Less than Actual” 24.4%), and the No Mirror Game Group (“Prediction Greater than Actual” 50%, “Prediction Equal to Actual” 11.6%, and “Prediction Less than Actual” 38.4%) exhibited significant differences, *χ*^2^ = 19.56, *p* < 0.001 (Figure 5).

**(3)**  **Aha! Experience Intensity: One-Way ANOVA and Bayesian estimation**

To investigate the differences in the intensity of the Aha! experience, the dependent variable “Aha! Experience Intensity” was measured using participants’ self-reported ratings, with the average score used as the final value.

Since the dependent variable is continuous and meets the assumption of normality, a one-way analysis of variance (ANOVA) was conducted, as shown in Table 3.

**Figure 6:**
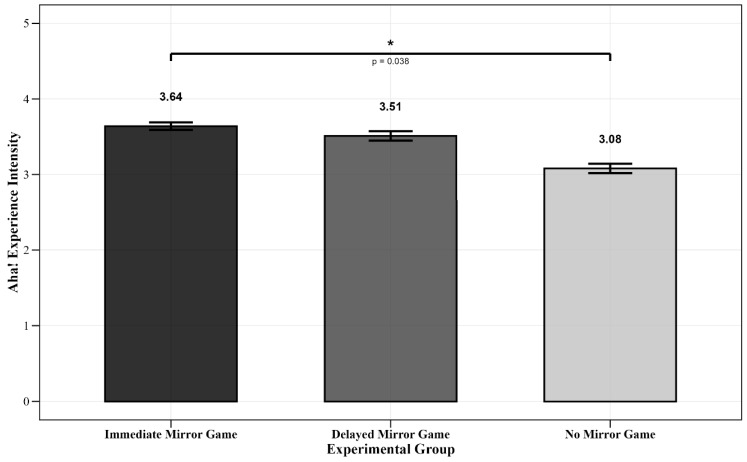
ANOVA statistical results for Aha! experience intensity. Note: Mean Aha! experience intensity scores across experimental groups. ANOVA results: *F*(2, 687) = 2.47, *p* = 0.097. Post-hoc comparison (LSD): Immediate Mirror Game > No Mirror Game, *p* = 0.038 *. Error bars represent the standard error of the mean. * *p* < 0.05.

Between-Group Comparison Results: A one-way ANOVA was conducted using the groups (Immediate Group, Delayed Group, No Mirror Game Group) as between-group variables to analyze differences in Aha! experience intensity. The analysis yielded *F*(2, 62) = 2.47, *p* = 0.097, *η_p_*^2^ = 0.07. Post-Hoc Multiple Comparisons: The post-hoc analysis showed that the Immediate Group (*M* = 3.64, *SD* = 0.77) had significantly higher Aha! experience intensity than the No Mirror Game Group (*M* = 3.08, *SD* = 0.90, Cohen’s *d* = 0.66), *p* = 0.038 (Figure 6).

**Figure 7:**
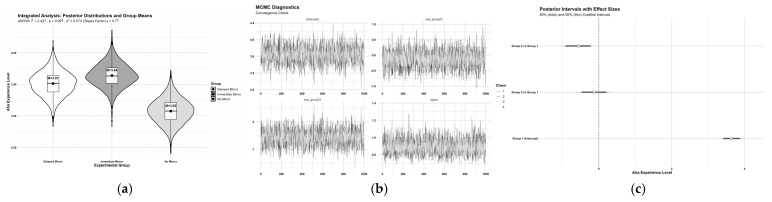
Bayesian results for Aha! experience intensity of group comparisons. Note: (**a**) Posterior distributions for Aha! experience intensity across the three experimental groups. Black dots represent posterior means; shaded violins represent estimated distributions. (**b**) Posterior differences between groups, displayed as effect sizes with 80% (thick) and 95% (thin) credible intervals. (**c**) MCMC diagnostics for model convergence, with four chains plotted per parameter. Overall model summary: F(2, 62) = 2.427, *p* = 0.097, *η_p_*^2^ = 0.073, Bayes factor_10_ = 0.77.

To strengthen the robustness of the analysis, Bayesian estimation was further applied to examine group differences in Aha! experience intensity, as shown in Table 3. Results from the posterior distributions indicated that the Immediate Mirror Group (*M* = 3.64, 95% CI [3.28, 4.02]) had the highest mean rating and served as the reference group. When compared with the Delayed Mirror Group (*M* = 3.51, 95% CI [2.98, 4.03]), the posterior effect size was −0.13, with 68.3% of the posterior distribution favoring the Immediate Group. This provided only weak evidence for the superiority of immediate mirror interaction over delayed mirror interaction. In contrast, comparison with the No Mirror Group (*M* = 3.08, 95% CI [2.54, 3.61]) yielded a larger effect size of −0.56, with 98.0% of the posterior distribution below zero. This result constitutes strong Bayesian evidence supporting the facilitative effect of immediate mirror interaction over no mirror interaction (Figure 7).

#### 3.1.2. Secondary Dependent Variables: Auxiliary Indicators of Aha! Experience Predictive Processing Performance

**(1)**  **Number of Solved Riddles, Correct Answers, and Aha! Experience Count: One-Way ANOVA**

To explore the differences in the number of solved riddles, correct answers, and the number of Aha! experiences in the insight puzzle task, a one-way ANOVA was conducted among the three groups (Immediate Group, Delayed Group, and No Mirror Game Group).

For the number of solved riddles, the group effect was not significant: Immediate Group (*M* = 7.83, *SD* = 2.12), Delayed Group (*M* = 7.38, *SD* = 2.34), and No Mirror Game Group (*M* = 7.76, *SD* = 2.28); *F*(2, 66) = 0.29, *p* = 0.752, *η_p_*^2^ = 0.01.

For the number of correct answers, the group effect was not significant: Immediate Group (*M* = 6.50, *SD* = 2.49), Delayed Group (*M* = 6.33, *SD* = 2.44), and No Mirror Game Group (*M* = 6.81, *SD* = 2.38); *F* (2, 66) = 0.22, *p* = 0.805, *η_p_*^2^ = 0.01.

For the number of Aha! experiences, the group effect was not significant: Immediate Group (*M* = 3.71, *SD* = 1.92), Delayed Group (*M* = 3.21, *SD* = 1.74), and No Mirror Game Group (*M* = 3.38, *SD* = 1.36); *F*(2, 57) = 0.53, *p* = 0.590, *η_p_*^2^ = 0.02.

These results indicate that there were no significant differences among the groups in the number of solved riddles, correct answers, and Aha! experiences, suggesting that the auxiliary indicators of Aha! experience predictive processing performance did not significantly affect the groups.

**(2)**  **Five Dimensions of Aha! Experience (Pleasure, Suddenness, Surprise, Confidence, and Impasse): One-Way ANOVA**

To examine whether the five dimensions of Aha! experience (Pleasure, Suddenness, Surprise, Confidence, and Impasse) significantly differed among the three groups (Immediate Group, Delayed Group, and No Mirror Game Group), a one-way ANOVA was conducted.

For the Pleasure dimension, the group effect was not significant: Immediate Group (*M* = 3.51, *SD* = 1.28), Delayed Group (*M* = 3.42, *SD* = 1.01), and No Mirror Game Group (*M* = 3.67, *SD* = 0.95); *F* (2, 61) = 0.28, *p* = 0.760, *η_p_*^2^ = 0.01.

For the Suddenness dimension, the group effect was not significant: Immediate Group (*M* = 3.05, *SD* = 0.98), Delayed Group (*M* = 2.86, *SD* = 1.14), and No Mirror Game Group (*M* = 2.93, *SD* = 0.65); *F* (2, 61) = 0.21, *p* = 0.813, *η_p_*^2^ = 0.01.

For the Surprise dimension, the group effect was not significant: Immediate Group (*M* = 3.06, *SD* = 1.12), Delayed Group (*M* = 3.24, *SD* = 0.95), and No Mirror Game Group (*M* = 3.04, *SD* = 0.92); *F* (2, 61) = 0.24, *p* = 0.791, *η_p_*^2^ = 0.01.

For the Confidence dimension, the group effect was not significant: Immediate Group (*M* = 4.48, *SD* = 0.70), Delayed Group (*M* = 4.34, *SD* = 0.81), and No Mirror Game Group (*M* = 4.51, *SD* = 0.78); *F* (2, 61) = 0.30, *p* = 0.744, *η_p_*^2^ = 0.01.

For the Impasse dimension, the group effect was not significant: Immediate Group (*M* = 3.05, *SD* = 1.06), Delayed Group (*M* = 2.92, *SD* = 1.19), and No Mirror Game Group (*M* = 2.83, *SD* = 0.89); *F* (2, 61) = 0.23, *p* = 0.793, *η_p_*^2^ = 0.01.

These results indicate that there were no significant differences among the three groups in the five dimensions of the Aha! experience, suggesting that the dimensions did not significantly influence the predictive processing performance of the Aha! experience.

#### 3.1.3. Additional Variables: Emotions

**(1)**  **Emotion (Pleasure, Arousal, and Control): One-Way ANOVA**

To investigate whether emotional states influenced the experimental results, a one-way ANOVA was conducted on the emotional states (Pleasure, Arousal, and Control) of the three groups (Immediate Group, Delayed Group, and No Mirror Game Group).

For emotional Pleasure, the group effect was not significant: Immediate Group (*M* = 3.38, *SD* = 2.00), Delayed Group (*M* = 2.92, *SD* = 1.53), and No Mirror Game Group (*M* = 3.52, *SD* = 1.75); *F* (2, 66) = 0.74, *p* = 0.483, *η_p_*^2^ = 0.022.

For emotional Arousal, the group effect was not significant: Immediate Group (*M* = 6.75, *SD* = 1.75), Delayed Group (*M* = 5.96, *SD* = 2.01), and No Mirror Game Group (*M* = 6.33, *SD* = 1.53); *F* (2, 66) = 1.18, *p* = 0.313, *η_p_*^2^ = 0.035.

For emotional Control, the group effect was not significant: Immediate Group (*M* = 3.00, *SD* = 2.09), Delayed Group (*M* = 3.33, *SD* = 1.86), and No Mirror Game Group (*M* = 2.71, *SD* = 1.79); *F*(2, 57) = 0.59, *p* = 0.560, *η_p_*^2^ = 0.017.

These results suggest that there were no significant differences in the emotional states of Pleasure, Arousal, and Control among the three groups, indicating that emotion did not significantly influence the experimental variables, and thus, the effect of emotions can be ruled out as a confounding factor.

### 3.2. Statistical Analysis of fNIRS Data

Following the preprocessing steps outlined in the systematic and methodology-focused review by Herold et al. (2018) on the applications of functional near-infrared spectroscopy (fNIRS) neuroimaging in exercise–cognition science, the data were processed as follows:

#### 3.2.1. Preprocessing

**(1)**  **Pruning Channels (hmrR_PruneChannels):**

Channels were automatically pruned based on distance range, signal-to-noise ratio threshold, and SD range.

**(2)**  **Converting Intensity to Optical Density (hmrR_Intensity2OD):**

The acquired data were converted from intensity values to optical density.

**(3)**  **Motion Artifact Correction (hmrR_MotionArtifactByChannel):**

Motion artifacts were automatically flagged using preset motion and mask time thresholds, as well as standard deviation and amplitude thresholds.

**(4)**  **Spline Motion Correction (hmrR_MotionCorrectSpline):**

Spline interpolation was used to correct motion artifacts identified during the previous step.

**(5)**  **Bandpass Filtering (hmrR_BandpassFilt):**

A bandpass filter (0.01–0.5 Hz) was applied using both high-pass and low-pass filters.

**(6)**  **Converting Optical Density to Concentration (hmrR_OD2Conc):**

Optical density data were converted to changes in oxygenated hemoglobin (HbO) and deoxygenated hemoglobin (HbR) concentrations using the modified Beer–Lambert law.

**(7)**  **CBSI Motion Correction (Cbsi_Motion_Correction):**

Motion correction was applied using a channel-based signal intensity correction method.

Since previous studies have demonstrated that oxygenated hemoglobin (HbO) is more sensitive to changes in blood flow, the present study focused exclusively on analyzing the concentration of HbO.

#### 3.2.2. Low-Frequency Amplitude

To assess the “relative activation” of the SCAN network, a one-way analysis of variance (ANOVA) was conducted on the fractional amplitude of low-frequency fluctuations (fALFF) during three task phases related to creativity (prediction of “solution time,” solving the “riddle task,” and self-assessment of the “Aha! experience”). Significant differences were found between the groups during the solution time prediction task.

**(1)**  **Predicted Solution Time: Significant differences among the three groups.**

The ANOVA *F*-test revealed that the SCAN network’s fALFF showed significant relative activation, as shown in Table 4.

**Figure 8:**
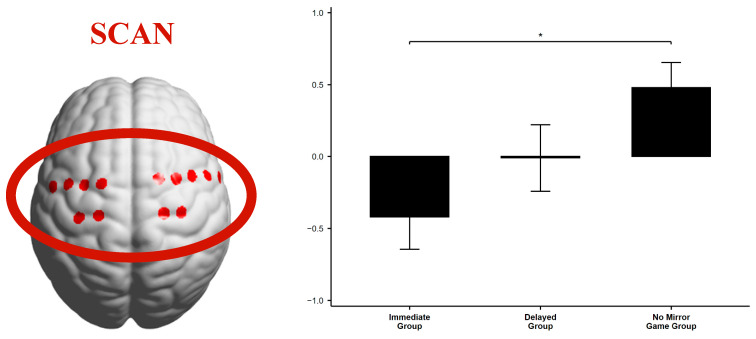
ANOVA *F*-test results for “Relative Activation” of the SCAN network during the predicted solution time task. Note: Asterisks indicate *p* < 0.05, representing significant differences between groups at the 0.05 level. Red bullets on the brain indicate the measurement channels positioned over the SCAN network regions.

Post hoc analysis with Bonferroni correction indicated that the Immediate Mirror Game group had significantly lower activation compared to the No Mirror Game group (*P*_Bonferroni_ = 0.0122) (Figure 8).

#### 3.2.3. Channel Functional Connectivity

To examine “channel functional connectivity,” Pearson correlation analyses were conducted between channel pairs during the five task phases of the experiment. The results showed that there were no significant functional connectivity differences during the Mirror Game learning phase, the formal Mirror Game phase, or the solution time prediction task. However, significant connectivity differences were observed during the riddle-solving task and the self-assessed Aha! experience task. Below, we report the significant results for these two tasks.

**(1)**  **Word Puzzle-Solving Task**

**Figure 9:**
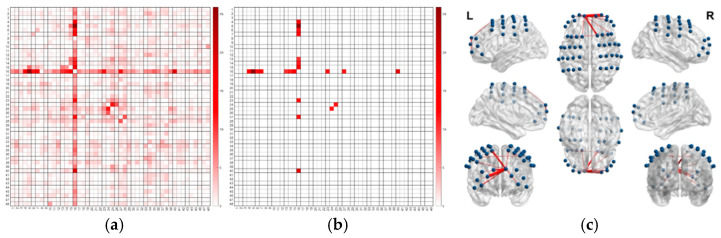
ANOVA *F*-test statistical results for channel functional connectivity during the word puzzle-solving task. Note: (**a**) Channel functional connectivity correlation matrix (uncorrected); (**b**) significant channel connectivity map (FDR corrected); (**c**) brain functional connectivity weight map (FDR corrected). Blue bullets indicate the measurement channels used for functional connectivity mapping.

The statistical results of channel functional connectivity during the Word Puzzle-Solving task are shown in Figure 9.

After conducting an ANOVA *F*-test, significant functional connectivity was found between the following channel pairs (with FDR correction applied): Ch16 and Ch4, Ch16 and Ch5, Ch16 and Ch6, Ch16 and Ch7, Ch16 and Ch13, Ch16 and Ch14, Ch16 and Ch15, Ch16 and Ch23, Ch16 and Ch27, Ch16 and Ch40, as well as Ch24 and Ch25, as shown in Table 5.

Further post-hoc analysis revealed that these differences primarily stemmed from the contrasts between the Immediate Group and the No Mirror Game Group, as well as between the Delayed Group and the no Mirror Game Group (with Bonferroni-corrected *p*-values all below 0.05). However, no significant differences were found between the Immediate and Delayed Groups.

**(2)**  **Self-Evaluated Aha! Experience Task**

**Figure 10:**
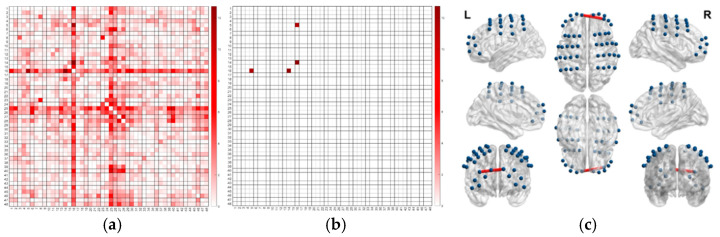
ANOVA *F*-test results for channel functional connectivity during the self-evaluated Aha! experience task. Note: (**a**) Channel functional connectivity correlation matrix (uncorrected); (**b**) significant channel functional connectivity (FDR-corrected); (**c**) brain functional connectivity weight map (FDR-corrected). Blue bullets indicate the measurement channels used for functional connectivity mapping.

The statistical results of channel functional connectivity during the Self-Evaluated Aha! Experience task are shown in Figure 10.

An ANOVA *F*-test revealed that the functional connectivity between Ch16 and Ch5, as well as between Ch16 and Ch14, was significant (FDR-corrected), as shown in Table 6.

Post-hoc analysis of Ch16 and Ch5 revealed that the Mirror Game Immediate Group showed significantly greater functional connectivity compared to the No Mirror Game group (*P*_Bonferroni_ = 0.004), and the Delayed Group also showed significantly greater connectivity compared to the No Mirror Game group (*P*_Bonferroni_ < 0.001). However, there was no significant difference between the Immediate and Delayed Groups.

Similarly, for Ch16 and Ch14, post-hoc analysis indicated that the Mirror Game Immediate Group had significantly higher functional connectivity than the No Mirror Game group (*P*_Bonferroni_ = 0.006), and the Delayed Group also showed significantly greater connectivity compared to the No Mirror Game group (*P*_Bonferroni_ < 0.001), with no significant difference observed between the Immediate and Delayed Groups.

**(3)**  **Channel Functional Connectivity Similarity**

Based on the results of channel functional connectivity, we further analyzed the similarity of channel functional connectivity between the formal stage of the Mirror Game and the three tasks related to insight experience. The results showed that the Spearman correlation coefficients for the similarity of channel functional connectivity were all greater than 0.7, indicating a high positive correlation, as shown in Table 7.

#### 3.2.4. Brain Network Functional Connectivity

Based on the results of the channel functional connectivity, it was found that Channel 16 (Ch16) in the RN network exhibited significant functional connectivity differences with many channels, while other channels in the RN network (channels 4, 5, 6, 7, 15) did not show significant differences. **This suggests that Ch16 may play a specific role, and therefore, further analysis of the RN brain network will focus on Ch16**.

To examine “brain network functional connectivity,” we performed Pearson correlation analyses among the three brain networks across the five task stages in the experiment, based on the experimental hypotheses. The results revealed significant functional connectivity during the Mirror Game learning stage, the word puzzle-solving task, and the self-reported Aha! experience task. However, no significant connectivity was observed during the formal stage of the Mirror Game. Below, we only report the following significant results: **(1) Dorsal Attention Network (DAN, FEF):** Channels 27, 28, 39, 40; **(2) Ventral Attention Network (VAN, DLPFC):** Channels 3, 8, 13, 14, 17, 18, 23, 24, 25, 26; **(3) Reward Circuit (RN, FPC):** Channel 16.

**(1)**  **Mirror Game Learning Stage**

**Figure 11:**
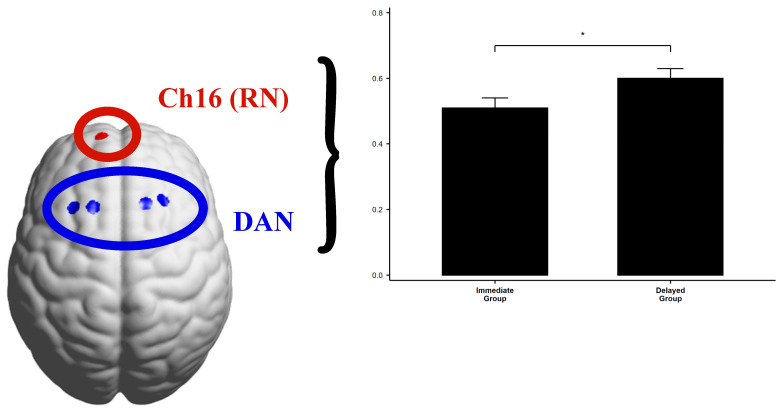
Statistical results of the independent samples *t*-test for brain network functional connectivity during the mirror game learning stage. Note: The functional connectivity between Ch16 and DAN shows that Ch16-DAN connectivity in the Immediate Group is significantly lower than that in the Delayed Group. Asterisks indicate *p* < 0.05, denoting significant group differences at the 0.05 level.

An independent sample *t*-test revealed that the functional connectivity between DAN and Ch16 was significantly lower in the Mirror Game Immediate Group compared to the Delayed Group (*p* = 0.034), as shown in Table 8 and Figure 11.

**(2)**  **Word Puzzle-Solving Task**

An ANOVA *F*-test revealed that the functional connectivity between DAN and Ch16, as well as between VAN and Ch16, was significant, as shown in Table 9.

**Figure 12:**
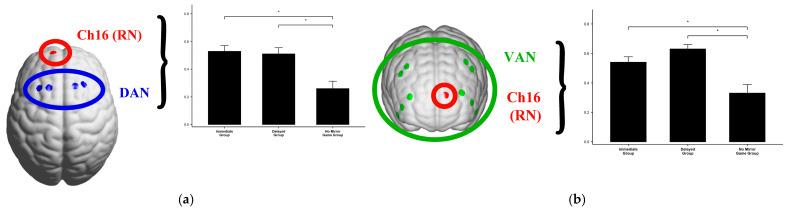
ANOVA *F*-test statistical results for brain network functional connectivity during the word puzzle-solving task. Note: (**a**) The figure shows the functional connectivity between DAN and Ch16, indicating that the Immediate Group is significantly higher than the No Mirror Group, and the Delayed Group is significantly higher than the No Mirror Group. (**b**) The figure shows the functional connectivity between VAN and Ch16, indicating that the Immediate Group is significantly higher than the No Mirror Group, and the Delayed Group is significantly higher than the No Mirror Group. Asterisks indicate *p* < 0.05, denoting significant differences between groups at the 0.05 level.

For the functional connectivity between DAN and Ch16, a post-hoc analysis with Bonferroni correction revealed that the Immediate Group showed significantly higher connectivity than the No Mirror Group (*P*_Bonferroni_ < 0.001), and the Delayed Group also showed significantly higher connectivity than the No Mirror Group (*P*_Bonferroni_ < 0.001), with no significant difference between the Immediate and Delayed Groups (Figure 12).

Similarly, for the connectivity between VAN and Ch16, the post-hoc analysis with Bonferroni correction revealed that the Immediate Group exhibited significantly higher connectivity than the No Mirror Group (*P*_Bonferroni_ = 0.003), and the Delayed Group also showed significantly higher connectivity than the No Mirror Group (*P*_Bonferroni_ < 0.001), with no significant difference between the Immediate and Delayed Groups (Figure 12).

**(3)**  **Self-Evaluated Aha! Experience Task**

An ANOVA *F*-test revealed significant functional connectivity results between DAN and Ch16, as well as VAN and Ch16 during the self-evaluated Aha! experience task, as shown in Table 10.

**Figure 13:**
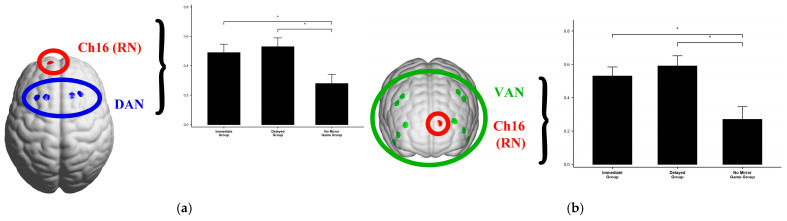
ANOVA *F*-test results for brain network functional connectivity during the self-evaluated Aha! experience task. Note: (**a**) Brain network functional connectivity between DAN and Ch16, indicating that the Immediate Group is significantly higher than the No Mirroring Group, and the Delayed Group is significantly higher than the No Mirroring Group. (**b**) Brain network functional connectivity between VAN and Ch16, indicating that the Immediate Group is significantly higher than the No Mirroring Group, and the Delayed Group is significantly higher than the No Mirroring Group. Asterisks represent *p* < 0.05, indicating significant differences between groups at the 0.05 level.

For the functional connectivity between DAN and Ch16, further post-hoc analysis with Bonferroni correction revealed that the Immediate Group showed significantly higher connectivity compared to the No Mirror Game Group (*P*_Bonferroni_ = 0.046), and the Delayed Group also showed significantly higher connectivity than the No Mirror Game Group (*P*_Bonferroni_ = 0.015). However, there was no significant difference between the Immediate and Delayed Groups (Figure 13).

Similarly, for the functional connectivity between VAN and Ch16, further post-hoc analysis with Bonferroni correction indicated that the Immediate Group exhibited significantly higher connectivity than the No Mirror Game Group (*P*_Bonferroni_ = 0.015), and the Delayed Group showed significantly higher connectivity compared to the No Mirror Game Group (*P*_Bonferroni_ = 0.003). There was no significant difference between the Immediate and Delayed Groups (Figure 13).

## 4. Discussion

### 4.1. Behavioral Results: Temporal Synchronization in Bodily Interaction Influences the Aha! Experience via Metacognitive Predictive Processing

This study builds upon the “Minimization of Metacognitive Prediction Error” model proposed by Dubey et al. (2021), by constructing a three-phase insight task paradigm—prediction, action, and evaluation—to examine whether temporal synchronization in bodily interaction influences the intensity of the Aha! experience through the modulation of metacognitive prediction error. Frequentist statistical analyses revealed significant or marginally significant differences among the three groups in predicted solution time, time prediction error, and Aha! ratings, suggesting that the temporal structure of physical interaction may indeed play a role in higher-order cognitive regulation.

First, in the predicted solution time, participants in both the Immediate and Delayed Mirror Groups exhibited more extreme time estimates. This pattern may stem from a transfer effect of motor rhythm formed during the mirror interaction, where participants projected their perception of “fast” or “slow” movements onto the subsequent time prediction task. Time prediction error also showed group differences, with the Immediate Mirror Group more likely to exhibit “overestimated” predictions—where predicted time exceeded actual time—consistent with Dubey et al.’s proposal that insight is more likely to occur when a problem is solved unexpectedly quickly. Finally, in terms of subjective Aha! ratings, the Immediate Mirror Group scored significantly higher than the No Mirror Group, supporting the behavioral hypothesis that high-synchrony interaction enhances insight experience.

To reinforce the robustness of the analysis, the study also employed Bayesian inference to supplement group-level comparisons. Posterior distribution results indicated a clear trend of higher Aha! ratings in the Immediate Mirror Group, not only distinguishing it from the No Mirror Group but also demonstrating greater consistency in effect strength and credibility. In contrast, differences between the Immediate and Delayed Mirror Groups were relatively modest, suggesting that bodily interaction with different temporal structures may differentially modulate the Aha! experience. The alignment between frequentist and Bayesian results offers dual validation for the hypothesis that synchronous physical interaction facilitates stronger insight experiences.

### 4.2. Low-Frequency Amplitude: Aha! Experience and the SCAN Network

Based on the theoretical hypotheses mentioned above, the Aha! experience is likely also associated with motor cortex activity, as indicated by SCAN network activation during insight tasks.

In terms of low-frequency amplitude results, ANOVA revealed significant differences in the relative activation of the SCAN network during the predicted solution time task. Specifically, the Immediate Mirror Game Group showed significantly lower fALFF values compared to the No Mirror Group. This is consistent with the behavioral results: in the first “predicted solution time” task, the Immediate Mirror Group chose “seconds” significantly more often than the other two groups, while the Delayed Group selected “minutes” more frequently. These findings suggest that prior mirror-based interactions, whether immediate or delayed, influenced participants’ subsequent prediction tasks, indicating that the SCAN network was involved in the predicted solution time task.

### 4.3. Channel Functional Connectivity: Ch16 in the Reward Circuit Plays a Key Role

First, the results from channel functional connectivity analysis showed significant group differences in the connectivity of channels 4, 5, 6, 7, 15, and 16 in the reward circuit RN (FPC), suggesting that Ch16 plays a pivotal role in the Aha! experience and metacognitive time prediction error.

A possible explanation is that the frontopolar cortex (FPC), particularly Brodmann area 10 (BA10), is considered a key brain region for metacognition (Gilbert et al. 2006). Metacognition involves monitoring and regulating one’s cognitive processes, including self-assessment, decision-making, problem-solving, and future scenario planning. In a review by Burgess et al. (2007), the FPC’s role in time management, planning, and decision-making is emphasized, particularly its importance in metacognitive control, which involves assessing oneself and the environment and adjusting behavior to achieve long-term goals. Ch16 appears to play a key role in the reward circuit RN, warranting further investigation into its specific function in this network.

Furthermore, the channel functional connectivity similarity results indicated high positive correlations (Spearman coefficient > 0.7) between the mirror game stage and insight tasks across all three tasks, suggesting that the temporal synchronization of physical interaction was applied in subsequent insight tasks. The neural mechanism linking physical interaction and the Aha! experience is likely rooted in metacognitive predictive processing.

### 4.4. Brain Network Functional Connectivity: The Role of RN, DAN, and VAN in Metacognitive Predictive Processing

Brain network functional connectivity results indicated that Ch16 was significantly connected to the dorsal attention network (DAN) and ventral attention network (VAN), while no significant differences were observed in the other RN channels (4, 5, 6, 7, 15). Several factors may explain this:

Firstly, the brain’s functional networks are dynamically coordinated, and interactions between different networks may become more active under specific conditions. Ch16 may be positioned at a crucial node that reflects this dynamic coordination, while other channels may not capture these interactions as effectively. Additionally, the experimental task may particularly rely on cognitive control and attentional mechanisms associated with the dorsal attention network (DAN) and ventral attention network (VAN). The location of Ch16 may be more directly involved in the cognitive processes required for solving riddles and evaluating Aha! experiences, whereas other channels within the reward circuit may contribute less to these specific tasks. Furthermore, differences in functional connectivity could reflect neuroanatomical specificity. The area around Ch16 may have more direct or critical connections to DAN and VAN due to its anatomical location, while other channels may be situated in regions that are relatively distant or less strongly connected.

In summary, based on the results from both channel and brain network functional connectivity analyses, the SCAN network (motor cortex) does not appear to be directly related to the Aha! experience during the riddle-solving and self-evaluated Aha! tasks. Instead, the findings point to a metacognitive predictive processing mechanism involving the dorsal attention network (DAN), the reward network (RN), and the ventral attention network (VAN).

This suggests a theoretical framework where the metacognitive predictive processing mechanism that governs the Aha! experience is mediated by the interaction between DAN, VAN, and RN. DAN primarily controls goal-directed, voluntary attention in visual spatial tasks (Corbetta and Shulman 2002). Depending on task demands, DAN coordinates with VAN (or the salience network) during task-switching or attention shifts (Tamber-Rosenau et al. 2018), while the SCAN network appears to have no role in this process.

## 5. General Conclusions and Future Directions

### 5.1. Theoretical Contributions: From Bodily Interaction to Insight—An Innovative Pathway Through Implicit Metacognitive Predictive Processing

#### 5.1.1. Theoretical Innovation: Proposal of the “Implicit Metacognitive Predictive Processing” Mechanism

Building upon the “minimization of metacognitive prediction error” framework (Dubey et al. 2021), this study introduces the novel concept of implicit metacognitive predictive processing. This mechanism suggests that insight experiences are not purely random events, but rather emerge from an implicit and dynamic system involving bodily perception, prediction deviation, and metacognitive feedback. Of particular note is the finding that temporal synchrony in bodily interaction significantly modulates individuals’ internal expectations of task efficiency. This modulation amplifies positive prediction errors upon successful problem-solving, thereby enhancing both the intensity and emotional salience of insight experiences. At a broader level, this model offers a theoretical bridge that integrates embodied cognition, enactive cognition, and metacognitive predictive processing, providing a cross-level and cross-system account of the cognitive mechanisms underlying insight.

#### 5.1.2. Methodological Innovation: Establishing a Three-Phase Paradigm for Metacognitive Predictive Processing

At the methodological level, this study constructs a systematic three-phase paradigm (Prediction–Execution–Evaluation) for eliciting insight, incorporating subjective temporal prediction, behavioral performance, and self-reported insight intensity. This design captures the full temporal dynamics of insight generation and provides a flexible, generalizable paradigm for future creativity research. Notably, the use of mirror-based bodily interaction as a pre-task manipulation illustrates that nonverbal, low-cost bodily coordination tasks can exert measurable effects on high-level cognition. This provides a methodological innovation and an empirical model for future embodied cognition experiments.

### 5.2. Empirical Limitations: Constraints and Areas for Refinement

Despite the experimental and methodological advances presented in this study, several limitations remain. First, although the sample size met the minimum requirements for statistical power, it was insufficient to capture individual differences in cognitive styles, motor fluency, or personality traits. Future studies should include larger and more diverse samples and incorporate trait-level variables to explore their moderating effects. Second, the neuroimaging analysis relied primarily on static measures of channel- and network-level functional connectivity. Without the integration of dynamic causal modeling or time-series analyses, the temporal causality within the predictive processing chain remains unexplored. Third, the riddle-solving task, while experimentally robust, offers limited ecological validity. Future research should explore more realistic problem-solving scenarios (e.g., interdisciplinary modeling, social decision-making) to better generalize the findings to real-world cognitive contexts.

### 5.3. Practical Implications: Cross-Disciplinary Applications and Future Potential

In education, elements such as mirror interaction and rhythmic synchrony can be embedded into collaborative learning designs to enhance students’ insight experiences, motivational engagement, and cognitive flexibility in complex problem-solving tasks. In creativity training and team collaboration, mirror-based or rhythm-synchronized “cognitive pre-activation” exercises (e.g., group rhythm imitation, co-movement, motor relay) can be integrated into corporate training and creative workshops to spark inspiration and foster emotional resonance, thereby enhancing collective creativity output. In neurotechnology and brain–computer interfaces, the dynamic patterns observed in the DAN–VAN–RN network—particularly the frontopolar cortex represented by Ch16—can serve as neural targets for modulating the “insight threshold.” This has implications for the development of neuroadaptive tools in cognitive enhancement, neural feedback, and real-time insight facilitation.

## Figures and Tables

**Table 1:** Chi-square test statistical analysis results for the composition ratio of predicted solution time.

Experimental Group	Total	In Seconds	In Minutes	Chi-Square Test	
	*n*	*n* (%)	*n* (%)	*χ* ^2^	*p*
Immediate Mirror Game Group	240	132 (55%)	108 (45%)	32.15	<0.001
Delayed Mirror Game Group	240	74 (30.8%)	166 (69.2%)		
No Mirror Game Group	210	106 (50.5%)	104 (49.5%)		

**Table 2:** Chi-square test statistical analysis results for the “Composition Ratio” of time prediction error.

Experimental Group	Total	Prediction Greater than Actual	Prediction Equals Actual	Prediction Less than Actual	Chi-Square Test	
	*n*	*n* (%)	*n* (%)	*n* (%)	*χ* ^2^	*p*
Immediate Mirror Game Group	182	92 (50.5%)	12 (6.6%)	78 (42.9%)	19.56	<0.001
Delayed Mirror Game Group	176	121 (68.8%)	12 (6.8%)	43 (24.4%)		
No Mirror Game Group	164	82 (50%)	19 (11.6%)	63 (38.4%)		

**Table 3:** Combined results of traditional and Bayesian analyses for Aha! experience intensity, including group means, ANOVA, *t*-tests, effect sizes, Bayes factors, and posterior probabilities.

**ANOVA**	**Experimental Group**	***M* (*SD*)**	***F*-Test**			**Multiple Comparisons**
			***F***	***p***	***η_p_* ^2^**	
	Immediate Mirror Game Group	3.64 (0.77)	*F*(2, 62) = 2.47	0.097	0.07	Immediate Mirror Game Group > No Mirror Game Group (*p* = 0.038)
	Delayed Mirror Game Group	3.51 (0.97)				
	No Mirror Game Group	3.08 (0.90)				
**BAYES**	**Experimental** **Group Comparison**	**t(df),** ***p*-Value**	**Cohen’s *d* [95% CI]**	**BF_10_**	**Bayesian Evidence**	**Posterior Probability**
	Immediate vs. Delayed	t(38) = 0.49, *p* = 1.000	0.15 [−0.46, 0.76]	0.33	Strong evidence for H_0_	68.3% (Immediate > Delayed)
	Immediate vs. No Mirror	t(40) = 2.22, *p* = 0.096	0.68 [0.05, 1.30]	2.12	Weak evidence for H_1_	98.0% (Immediate > No Mirror)
	Delayed vs. No Mirror	t(40) = 1.50, *p* = 0.421	0.46 [−0.17, 1.10]	0.74	Moderate evidence for H_0_	94.3% (Delayed > No Mirror)

**Table 4:** ANOVA *F*-test results for the “Relative Activation” of the SCAN network during the predicted solution time task.

Brain Network	Experimental Group	Descriptive Statistics			*F*-Test		
		*M*	*SD*	*N*	*F* (df1, df2)	*p*	*η_p_* ^2^
SCAN	Immediate Group	−0.42	1.03	21	*F*(2, 56) = 4.50	0.015	0.14
	Delayed Group	−0.01	1.01	19			
	No Mirror Game Group	0.48	0.76	19			

**Table 5:** Descriptive statistics for significant channel functional connectivity during the word puzzle-solving task.

Channel Functional Connectivity	Experiment Group	Descriptive Statistics			*F*-Test		
		*M*	*SD*	*N*	*F* (df1, df2)	*p*	*η_p_* ^2^
Ch16 and Ch4	Immediate	0.57	0.23	21	*F*(2, 56) = 13.80	<0.001	0.33
	Delayed	0.59	0.21	19			
	No Mirror	0.24	0.26	19			
Ch16 and Ch5	Immediate	0.74	0.19	21	*F*(2, 56) = 25.90	<0.001	0.481
	Delayed	0.80	0.26	19			
	No Mirror	0.28	0.28	19			
Ch16 and Ch6	Immediate	0.53	0.23	21	*F*(2, 56) = 11.84	<0.001	0.297
	Delayed	0.62	0.24	19			
	No Mirror	0.25	0.26	19			
Ch16 and Ch7	Immediate	0.76	0.22	21	*F*(2, 56) = 12.27	<0.001	0.305
	Delayed	0.86	0.23	19			
	No Mirror	0.44	0.36	19			
Ch16 and Ch13	Immediate	0.52	0.23	21	*F*(2, 56) = 10.30	<0.001	0.269
	Delayed	0.59	0.23	19			
	No Mirror	0.27	0.24	19			
Ch16 and Ch14	Immediate	0.62	0.33	21	*F*(2, 56) = 11.32	<0.001	0.288
	Delayed	0.78	0.26	19			
	No Mirror	0.34	0.26	19			
Ch16 and Ch15	Immediate	0.76	0.40	21	*F*(2, 56) = 16.39	<0.001	0.369
	Delayed	0.90	0.24	19			
	No Mirror	0.34	0.27	19			
Ch16 and Ch23	Immediate	0.46	0.31	21	*F*(2, 56) = 11.80	<0.001	0.296
	Delayed	0.52	0.26	19			
	No Mirror	0.13	0.22	19			
Ch16 and Ch27	Immediate	0.52	0.30	21	*F*(2, 56) = 11.71	<0.001	0.295
	Delayed	0.53	0.22	19			
	No Mirror	0.18	0.23	19			
Ch16 and Ch40	Immediate	0.57	0.26	21	*F*(2, 56) = 18.92	<0.001	0.403
	Delayed	0.53	0.23	19			
	No Mirror	0.17	0.15	19			
Ch24 and Ch25	Immediate	0.62	0.30	21	*F*(2, 56) = 11.63	<0.001	0.293
	Delayed	0.70	0.25	19			
	No Mirror	0.31	0.24	19			

**Table 6:** Descriptive statistics for channel functional connectivity between Ch16 and Ch5, Ch16 and Ch14, during the self-evaluated Aha! experience task.

Channel Functional Connectivity	Experiment Group	Descriptive Statistics			*F*-Test		
		*M*	*SD*	*N*	*F* (df1, df2)	*p*	*η_p_* ^2^
Ch16 and Ch5	Immediate	0.60	0.32	21	*F*(2, 56) = 11.41	<0.001	0.290
	Delayed	0.75	0.39	19			
	No Mirror	0.24	0.30	19			
Ch16 and Ch14	Immediate	0.52	0.36	21	*F*(2, 56) = 12.74	<0.001	0.313
	Delayed	0.74	0.45	19			
	No Mirror	0.14	0.27	19			

**Table 7:** Similarity of channel functional connectivity between the formal mirror game experiment and the three insight tasks.

Task 1	Task 2	Experimental Group	Channel Functional Connectivity Similarity
Formal Experiment Stage of the Mirror Game	Predicted Solution Time	Immediate Group	0.72
		Delayed Group	0.75
	Solved Word Puzzle Task	Immediate Group	0.80
		Delayed Group	0.87
	Self-Evaluated Aha! Experience	Immediate Group	0.73
		Delayed Group	0.80

**Table 8:** Descriptive statistics of the functional connectivity between DAN and Ch16 during the mirror game learning stage.

Brain Network Functional Connectivity	Experiment Group	*M*	*SD*	*N*	t(df)	*p*	Cohen’s *d*
DAN and Ch16	Immediate Group	0.51	0.14	21	t(38) = −2.20	0.034	−0.696
	Delayed Group	0.60	0.13	19			

**Table 9:** ANOVA *F*-test statistical results for brain network functional connectivity during the word puzzle-solving task.

Brain Network Functional Connectivity	Experiment Group	*M*	*SD*	*F* (df1, df2)	*p*	*η_p_* ^2^
DAN and Ch16	Immediate	0.53	0.19	*F*(2, 56) = 10.75	<0.001	0.278
	Delayed	0.51	0.20			
	No Mirror	0.26	0.23			
VAN and Ch16	Immediate	0.54	0.17	*F*(2, 56) = 12.61	<0.001	0.310
	Delayed	0.63	0.13			
	No Mirror	0.33	0.26			

**Table 10:** ANOVA *F*-test results for brain network functional connectivity during the self-evaluated Aha! experience task.

Brain Network Functional Connectivity	Experiment Group	*M*	*SD*	*F* (df1, df2)	*p*	*η_p_* ^2^
DAN and Ch16	Immediate	0.49	0.26	*F*(2, 56) = 4.97	0.010	0.151
	Delayed	0.53	0.26			
	No Mirror	0.28	0.27			
VAN and Ch16	Immediate	0.53	0.25	*F*(2, 56) = 7.07	0.002	0.202
	Delayed	0.59	0.27			
	No Mirror	0.27	0.33			

## Data Availability

The data supporting the findings of this study are hosted on the Open Science Framework (OSF) platform. However, access to the data is restricted to safeguard participant privacy. Researchers interested in accessing the dataset may contact the first author (Jiajia Su) to request permission. Data will be shared upon reasonable request and approval.
